# Efficacy of rikkosan for primary burning mouth syndrome: a retrospective study

**DOI:** 10.1186/s13030-021-00221-2

**Published:** 2021-10-30

**Authors:** Hiroyuki Hato, Ken-ichiro Sakata, Jun Sato, Takuya Asaka, Noritaka Ohga, Yutaka Yamazaki, Yoshimasa Kitagawa

**Affiliations:** 1 Department of Oral Diagnosis and Medicine, Faculty of Dental Medicine, Hokkaido University, Kita-13 Nishi-7, Kita-ku, 060-8586, Sapporo, Japan; 2 Gerodontology, Department of Oral Health Science, Faculty of Dental Medicine, Hokkaido University, Sapporo, Japan

**Keywords:** Burning mouth syndrome, Pharmacotherapy, Kampo medicine

## Abstract

**Background:**

Burning mouth syndrome (BMS) is a chronic condition characterized by pain in the oral cavity. Kampo medicine is a traditional Japanese medical system that has its roots partly in ancient Chinese medicine. The purpose of this study is to evaluate the efficacy of rikkosan—a traditional Japanese herbal medicine (Kampo)—in the treatment of primary BMS.

**Main body:**

A single-center retrospective study was conducted in 32 patients who were diagnosed with primary BMS and treated with rikkosan alone through gargling (2.5 g rikkosan dissolved in 50 mL hot water) three times daily. Patients were asked to evaluate their pain using a numerical rating scale (NRS) at first visit and after 1 month. One patient had stomatitis as a side effect after gargling with rikkosan, however, no side effects were observed in other patients. Overall NRS scores decreased significantly between the first visit (7.6 ± 2.7) and the 1-month visit (5.6 ± 2.8).

**Conclusions:**

Rikkosan may be an effective treatment for primary BMS.

## Background

Burning mouth syndrome (BMS) is a chronic pain characterized by a burning sensation in oral mucosal surfaces without related objective findings. Although several factors could be involved, including psychopathological factors and hormonal changes, the etiology of BMS remains unclear [1]. BMS tends to be classified as primary or secondary; primary BMS is where no dental or medical cause can be identified [1].

The treatment of primary BMS is mainly based on pharmacotherapy, brief psychotherapy, and cognitive behavioral therapy [1]. Previous systematic reviews have reported the efficacy of topical application of capsaicin [2] and topical and systemic administration of clonazepam [3]. Randomized controlled trials have reported the efficacy of systemic administration of paroxetine, sertraline, and alpha-lipoic acid [2, 3]. However, there is no concensus therapeutic intervention for primary BMS, and the above agents have been associated with side effects such as dizziness and drowsiness [3, 4]; therefore, careful administration is required.

Kampo medicine is a traditional Japanese medical system that has its roots partly in ancient Chinese medicine. Rikkosan is a traditional Japanese herbal medicine (Kampo) used to reduce oral pain caused by tooth decay, pulpitis, dentin hypersensitivity, periodontitis, stomatitis, and pain after tooth extraction [5–7]. Rikkosan consists of five crude herbs: *Asiasarum* root (saishin), *Cimicifuga* rhizome (shoma), *Saposhnikovia* root (boufu), *Glycyrrhiza* (kanzou) and Japanese Gentian (ryutan) [8]. It is believed to provide analgesia through a mechanism different to that of nonsteroidal anti-inflammatory drugs [6]. Although the effectiveness of rikkosan against BMS has been previously reported and several studies have addressed its efficacy for BMS [8, 9], few clinical studies have been conducted to evaluate its efficacy in a sufficient number of patients. This study aims to evaluate the effectiveness of rikkosan for primary BMS.

## Main text

## Methods

### Diagnostic algorithm

**Fig. 1:**
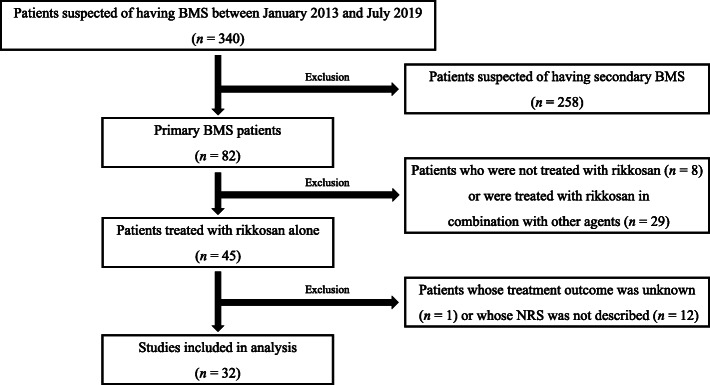
Our department’s diagnostic algorithm for primary burning mouth syndrome (BMS). Patients were diagnosed with primary BMS if they had residual symptoms after antifungal therapy, replacement therapy for deficiency factors, such as trace metals and vitamin B12, and after blood tests were normal

Patients suspected of BMS were examined and diagnosed in our department according to the algorithm shown in Fig. 1 and the criteria of The International Classification of Headache Disorders (ICHD-3) [10]. Patients were considered to have secondary BMS if systemic and psychosocial factors associated with secondary BMS and structural disorder in the oral cavity were found during examination. Patients with residual symptoms after antifungal therapy and replacement therapy for deficiency factors such as trace metals and vitamin B12 were diagnosed as primary BMS when blood tests were normal.

This retrospective study was conducted with the approval of the Hokkaido University Hospital Independent Clinical Research Review Committee (Approval No. 019-0044).

### Patients

**Fig. 2:**
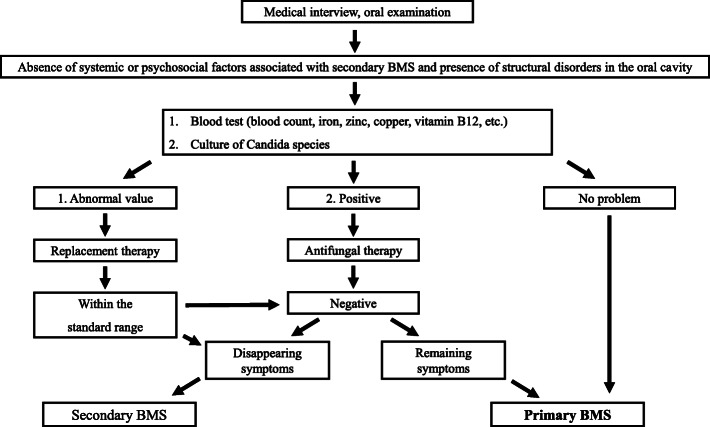
The inclusion and exclusion criteria. The study included 32 patients

The data of 340 patients who were suspected to have primary or secondary BMS were reviewed at the Department of Oral Medicine, Hokkaido University Hospital between January 2013 and July 2019 (Fig. 2). Patients with an underlying medical condition, based on interview, or any abnormalities found through our diagnostic algorithm were excluded (*n* = 258) because of possible secondary BMS. The details of secondary BMS as classified according to the classification proposed by Balasubramaniam et al. [11] are as follows: local factors for 40, systemic factors for 183, psychosocial factors for 51. Some patients had multiple factors, and 15 and 20 patients were diagnosed with secondary BMS caused by oral candidiasis and trace metal deficiency, respectively. Patients were excluded if rikkosan was not used (*n* = 8) or they were treated with rikkosan in combination with other agents (*n* = 29). Patients were excluded whose treatment outcome was unclear (*n* = 1) or whose numerical rating scale (NRS) score was not described (*n* = 12). Thirty-two patients who had clear medical records of their treatment were enrolled in this study.

### Treatment algorithm for primary BMS

We first explained to each participant the pathology of primary BMS. After explaining that there was no need for immediate surgical treatment and that the disease is non-fatal, we initiated pharmacotherapy according to each patient’s preference and did follow-ups once or twice a month. In our department, we often choose Japanese Kampo medicines as single agents for the initial pharmacotherapy because they have relatively few side effects and are easy to administer to elderly people [12]; however, anxiolytics and antidepressants may be ultimately selected for treatment based on the patient’s preference.

### Treatment dosage and administration

In this study, we administered single-agent rikkosan as a mouthwash for gargling (2.5 g rikkosan [Tsumura, Tokyo, Japan] dissolved in 50 mL hot water) three times daily (7.5 g/day).

### Study variables

Various factors, such as patient characteristics (age, sex) and clinical parameters (duration of illness, site of BMS, dosing period, treatment outcome, and side effects), were retrospectively examined. The duration of the disease was defined as the time from when the patient became aware of the symptoms to when the patient visited our department.

### Evaluation criteria of the therapeutic effects

The effectiveness of the treatment was assessed by referring to changes in NRS scores. NRS scores were evaluated by asking patients to assess the degree of pain they were currently experiencing, with 0 being no pain and 10 being the worst possible pain. NRS scores were measured at the time of the first visit and at 1 month after the initiation of gargling with rikkosan.

### Statistical analysis

Statistical analyses were performed using JMP Pro Version 14.0 (SAS Institute, Cary, NC) and included Wilcoxon rank test. Wilcoxon rank test was performed to assess significant differences in mean NRS scores at different time intervals. *P* < 0.05 was considered statistically significant.

## Results

The study sample consisted of 32 patients with primary BMS (30 female; mean age, 56 years; median, 55 years; range, 35–75 years). The mean duration of illness was 25 months (median, 7 months; range, 4–252 months). All patients had BMS that occurred on the tongue. The overall mean dosing period of rikkosan was 89 days (median, 85 days; range, 14–246 days).

**Fig. 3:**
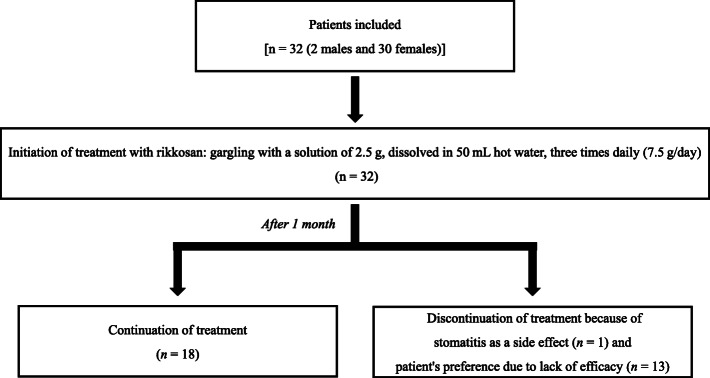
Flowchart demonstrating the treatment protocol followed by the patients. Fourteen patients discontinued the use of the agent after one month of treatment

**Fig. 4:**
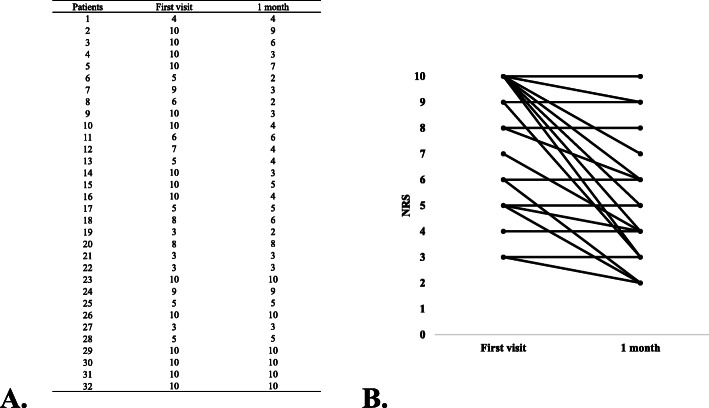
Changes in NRS scores between the first visit and one month after the initiation of gargling with rikkosan. **A** and (**B**) show the changes in pain scores for each patient treated with rikkosan

The outcomes of the treatment are shown in Figs. 3 and 4. Although one patient had stomatitis as a side effect after gargling with rikkosan, no other side effects were observed. Thirteen patients requested a change in the treatment agent at the one-month visit because they believed that rikkosan lacked efficacy. However, the overall NRS scores were significantly reduced, from 7.6 ± 2.7 at the first visit to 5.6 ± 2.8 at the 1-month visit (*P* < 0.05).

## Discussion

This is the first study to use NRS scores to assess the efficacy of rikkosan for primary BMS. Rikkosan treatment showed a significant reduction in NRS scores for primary BMS and did not show any serious side effects. These results indicate that gargled rikkosan could be a potential therapeutic option for primary BMS.

Anti-anxiety and antidepressant medications have been used for the treatment of BMS and their efficacy has been reported [2, 13, 14]. However, these agents are often associated with side effects such as dizziness and drowsiness [3, 4]. Side effects of medications must be considered when administering pharmacotherapy for BMS. In the present study, no obvious side effects such as those mentioned above were observed with rikkosan. In addition, since it is used as a gargle, it can be used as an alternative for patients who are resistant to the use of anxiolytics and antidepressants, and even for the elderly.

Although there are few studies on the pharmacological effects of rikkosan, some reports are available. Horie et al. [15] suggest that rikkosan inhibits prostaglandin E_2_ (PGE-2) production by selectively inhibiting cyclooxygenase-2 (COX-2) activity in activated macrophages in vivo. However, the pharmacological mechanism of pain control by rikkosan is still unclear; therefore, its mechanism of action in BMS remains unclear. Some of the crude herbs in rikkosan, particularly, *Asiasarum* root (saishin), are thought to have surface anesthetic action [9]. In BMS patients, transient receptor potential vanilloid 1 (TRPV1), a capsaicin receptor that responds to nociceptive stimulation, such as thermal stimulation, is upregulated in the lingual mucosal epithelium [16]. The components of saishin include agonists of TRPV1, such as methyl eugenol and higenamine (an alkaloid). It is our firm belief that some of the components of saishin bind to the TRPV1 receptor and produce an efficacy similar to topical capsaicin [13], although this is difficult to identify.

This study has several limitations. The number of overall cases and agents administrated was limited, in part because the target population was selected from patients with a diagnosis of primary BMS, rather than from a population who had used rikkosan. Definitive criteria for diagnosis of primary BMS have not yet been standardized; the definition used in this study may differ from that used in other studies. In addition, it is unclear if the effect of rikkosan is temporary or permanent because the patients have not been followed for an adequate time to determine a long-term recurrence rate. Furthermore, this study is not a randomized controlled trial but a retrospective study. It will be necessary to do further study that takes these factors into consideration.

## Conclusions

In summary, the results of our study suggest that rikkosan, a Japanese traditional Kampo medicine, can be effective in reducing the symptoms of patients with primary BMS.

## Data Availability

We are not able to share our data because sharing data is not permitted by our hospital or the ethics committee.

## Abbreviations

NRS: numerical rating scale
BMS: burning mouth syndrome
ICHD-3: The International Classification of Headache Disorders
PGE2: prostaglandin E2
TRPV1: transient receptor potential vanilloid 1
